# An Ethnographic Analysis of The Black and African American Adult Male Perspective on Alzheimer’s Disease and the Social Determinants of Health

**DOI:** 10.1002/alz.089439

**Published:** 2025-01-09

**Authors:** Lilcelia A. Williams, Jennifer H Lingler, Melita H Terry

**Affiliations:** ^1^ University of Pittsburgh Alzheimer’s Disease Research Center (ADRC), Pittsburgh, PA USA; ^2^ Department of Occupational Therapy, University of Pittsburgh, School of Health and Rehabilitation Sciences, Bridgeside Point I 100 Technology Drive, PA USA; ^3^ Department of Medicine Division of Geriatric Medicine, University of Pittsburgh, School of Medicine, Pittsburgh, PA USA; ^4^ University of Pittsburgh, Pittsburgh, PA USA

## Abstract

**Background:**

Black and African American adult males have the worst overall health than any other race or gender in the United States. The rate of Alzheimer’s Disease is twice as high for Black and African American adults. Yet, little is known about how Black and African American adult males perceive the relationship between the social determinants of health and Alzheimer’s Disease.

**Method:**

We conducted a secondary analysis of ethnographic interview data from ten Black and African American adult male participants at an NIA‐funded Alzheimer’s Disease Research Center. Participants included both patients (n = 6) and study partners (n = 4) who were purposively sampled to ensure that a range of perspectives were represented in the parent study, Recruitment Innovations for Diversity Enhancement (RIDE) in Alzheimer’s Disease Research. Chronological age and education were self‐reported. For the present analysis, transcripts from semi‐structured participant interviews were first examined to organize the data within categories informed the Social Determinants of Health Model. Data within each social determinant of health category were then analyzed to identify themes and explore potential relationships within the data.

**Result:**

All participants identified as male with a mean age of 75 (SD±13); 90% reported two or more years of post‐secondary education and 100% spontaneously mentioned elements of social determinants of health in response to questions about their experiences with dementia and motivations for participation in Alzheimer’s Disease research. A relationship between Alzheimer’s Disease and the social determinants of health was demonstrated by the study participants’ endorsement in across all domains. Locus of control, perception is reality, mistrust and othering are the thematic codes that consistently emerged from both the patient and caregiver interviews. The importance of having shared social characteristics (e.g., race) with clinicians, providers and staff members was a recurrent theme among the participants.

**Conclusion:**

Despite the shared social characteristics of race and gender among the study participants, their perception of the relationship between Alzheimer’s disease and the social determinants of health was not monolithic. Findings support the need for additional research to examine the perceptions and lived experiences of Black and African American adult males as it relates to Alzheimer’s Disease.

